# Plasma Dermatology: Skin Therapy Using Cold Atmospheric Plasma

**DOI:** 10.3389/fonc.2022.918484

**Published:** 2022-07-12

**Authors:** Fei Tan, Yang Wang, Shiqun Zhang, Runying Shui, Jianghan Chen

**Affiliations:** ^1^ Department of Otorhinolaryngology and Head & Neck Surgery (ORL-HNS), Shanghai Fourth People’s Hospital, and School of Medicine, Tongji University, Shanghai, China; ^2^ The Royal College of Surgeons in Ireland, Dublin, Ireland; ^3^ The Royal College of Surgeons of England, London, United Kingdom; ^4^ Department of Pathology, Shanghai Fourth People’s Hospital, and School of Medicine, Tongji University, Shanghai, China; ^5^ Department of Pharmacology, Shanghai Tenth People’s Hospital, and School of Medicine, Tongji University, Shanghai, China; ^6^ Department of Surgery, Department of Dermatology, Huadong Hospital, Fudan University, Shanghai, China; ^7^ Department of Surgery, Department of Dermatology, Shanghai Fourth People’s Hospital, and School of Medicine, Tongji University, Shanghai, China

**Keywords:** cold atmospheric plasma, medicine, skin, wound healing, skin cancer, dermatology, dermatitis, fungal infection

## Abstract

Cold atmospheric plasma-based plasma medicine has been expanding the diversity of its specialties. As an emerging branch, plasma dermatology takes advantage of the beneficial complexity of plasma constituents (e.g., reactive oxygen and nitrogen species, UV photons, and electromagnetic emission), technical versatility (e.g., direct irradiation and indirect aqueous treatment), and practical feasibility (e.g., hand-held compact device and clinician-friendly operation). The objective of this comprehensive review is to summarize recent advances in the CAP-dominated skin therapy by broadly covering three aspects. We start with plasma optimisation of intact skin, detailing the effect of CAP on skin lipids, cells, histology, and blood circulation. We then conduct a clinically oriented and thorough dissection of CAP treatment of various skin diseases, focusing on the wound healing, inflammatory disorders, infectious conditions, parasitic infestations, cutaneous malignancies, and alopecia. Finally, we conclude with a brief analysis on the safety aspect of CAP treatment and a proposal on how to mitigate the potential risks. This comprehensive review endeavors to serve as a mini textbook for clinical dermatologists and a practical manual for plasma biotechnologists. Our collective goal is to consolidate plasma dermatology’s lead in modern personalized medicine.

## 1 Plasma and Plasma Medicine

### 1.1 Cold Atmospheric Plasma

Depending on different context, plasma can be referred to as either the largest part of human blood (physiological plasma) or the fourth state of matter (physical plasma). In physics, plasma consists of electrons, ions, photons, metastables, and electromagnetic fields. Plasma was historically generated at high temperature and under high pressure. Nowadays, cold atmospheric plasma (CAP) could be produced at atmospheric pressure and operate under room temperature.

**Figure 1:**
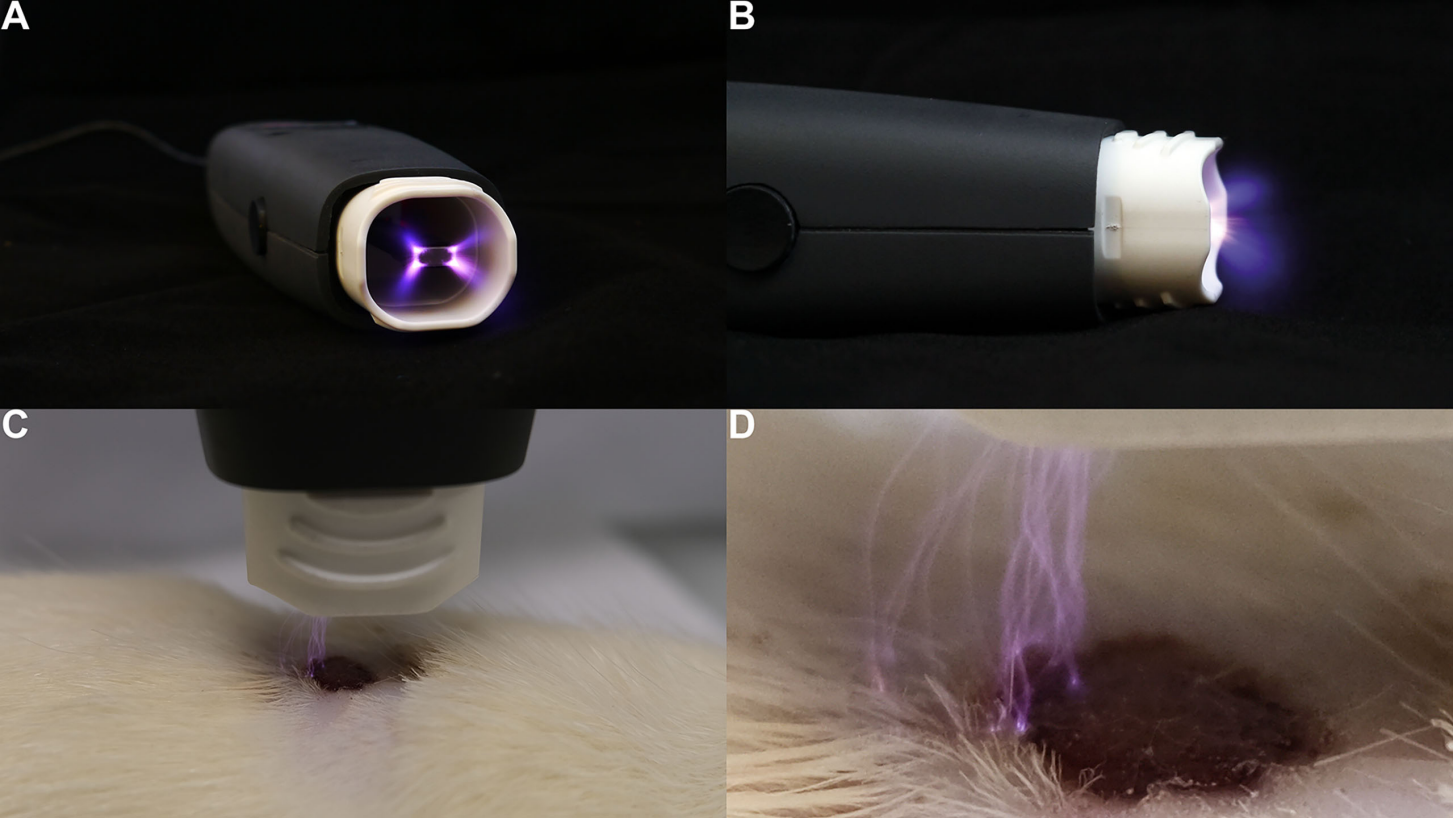
Demonstration of a hand-held CAP generator and its direct application on the skin. The PDD (piezoelectric direct discharge) technology-based plasma instrument, PZ3, was used to generate plasma on the cutaneous wound of a Sprague-Dawley rat’s dorsum (4). **(A)** axial view of the PZ3 outlet. **(B)** sagittal view of the PZ3 body. **(C)** Skin therapy using PZ3. **(D)** Close-up of PZ3 in operating mode.

Various technologies have been used to form atmospheric plasma (1, 2), including, but are not limited to, pulsed atmospheric arc (PAA) technology and piezoelectric direct discharge (PDD) technology (3). For example, a PDD CAP generator (**Figure 1**) uniquely combines voltage transformation and plasma generation in a single, compact, and highly efficient component (4). Its piezoelectric radiofrequency (RF) plasma generator provides several advantages, such as a high ionization speed, multi-gas ignition, low power consumption, and an effective generation rate of reactive oxygen and nitrogen species (RONS).

### 1.2 Plasma Medicine

The RONS produced by CAP’s hierarchical reaction with ambient air play a key role in various biological and cellular pathways, which has facilitated the application of CAP toward the medical field. In addition, the UV light and transient electric fields might also be important for biomedical applications (5–7). Plasma medicine, a newly coined term, implies an interdisciplinary subject incorporating physics, chemistry, life science, and medicine. The applications of plasma medicine can be broadly divided into direct ones and indirect ones. The former refers to exposing a living recipient under a gaseous plasma plume, and the latter implies delivering the reactive species of the CAP to a living recipient through an intermediate carrier.

The direct medical applications of CAP have been witnessed in several medical specialties. These include, but are not limited to, dentistry (e.g., tooth bleaching and root canal disinfection) (8), neurology (e.g., enhancing neural cell differentiation into neurons both *in vitro* and *in vivo*) (9), oncology (e.g., inducing immunogenic cancer cell death and synergy with other anti-neoplastic agents) (10), and infection control (e.g., controlling microbial biofilm) (11), etc. On the other hand, the indirect applications of CAP are achieved through either a solid object (e.g., a surgical implant) (12), a liquid object (e.g., the plasma-activated medium) (13), or a mixture (e.g., stem cell niche) (3). Irrespective of the operating mode, the biomedical effect of CAP penetrates through cellular, protein, and even DNA/mRNA levels (10, 13–15).

### 1.3 Plasma Dermatology

Plasma dermatology has emerged as an attractive specialty of plasma medicine, partially because skin is the largest and most superficial organ in the human body, which makes plasma treatment relatively easy (**Figure 1**). The original series of clinical randomized controlled trials (RCTs) of using CAP in dermatology focused primarily on chronic wounds (16, 17). Their success was multifactorial (18). Firstly, chronic wounds are a common skin condition, especially in the aging population and immunocompromised patients. Secondly, the treatment modalities for this disease are still quite limited. Lastly, the antimicrobial and anti-inflammatory features of CAP make it an ideal tool for a chronically colonised wound.

Since then, several reviews have been published on this topic, but each with pros and cons (19–24). Some are oversimplified in terms of disease spectrum, others failed to balance the clinical findings and scientific interpretation. Nonetheless, this is the first relevant review that is comprehensive, up-to-date, disease-specific, and bridging the gap between biotechnology and clinical practice. Literatures from only the last decade were extracted from PubMed and presented here in three aspects: optimising intact skin, treating skin disease, and improving drug absorbance into skin.

## 2 Optimising Intact Skin Using CAP

### 2.1 Clinical Anatomy of Human Skin

**Figure 2:**
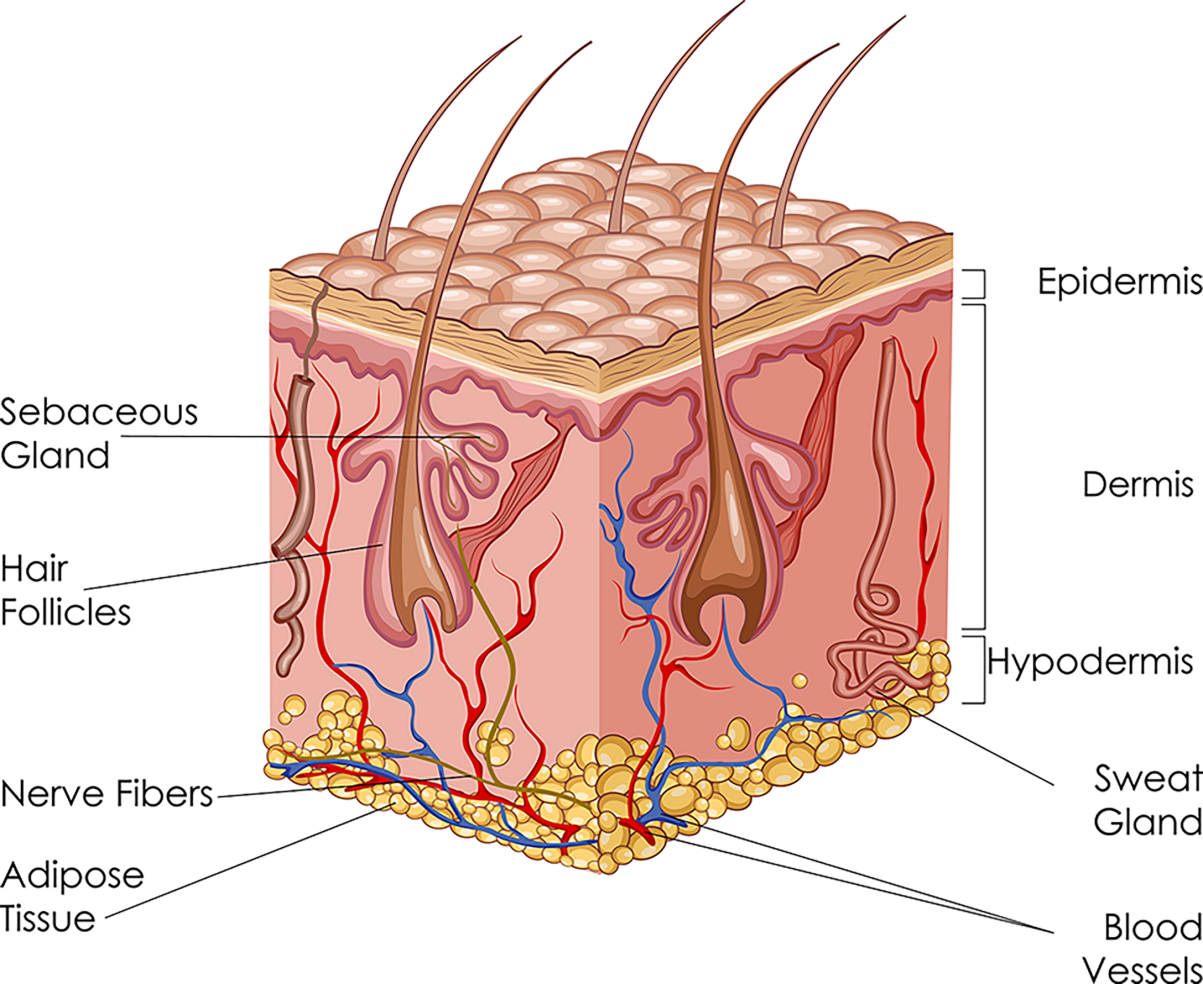
Schematic diagram of the skin: its three layers and accessory structures.

Human skin is a unique structure, with a surface area of 2 m^2^ and makes up for up to 20% of the total body weight. Skin is multifunctional as it provides protective barrier, acts as sensory receptor, transports nutrients and metabolites, helps regulate body temperature, and exercises immunological activity. Skin is composed of three layers, from superficial to deep: the epidermis, dermis, and hypodermis (**Figure 2**).

The epidermis is further divided into five sublayers: stratum corneum, stratum lucidum, stratum granulosum, stratum spinosum, and stratum basale. The basic cell types of epidermis include keratinocytes, melanocytes, and Langerhans cells. Keratinocytes are of ectodermal origin and have the specialized function of producing keratin. Melanocytes derive from the neural crest and synthesize melanin. Langerhans cells are dendritic cells and can recognize, process and present antigens to lymphocytes during hypersensitivity reaction.

The dermis is connected to the epidermis through the basement membrane zone. The dermis is divided into two areas: papillary region (superficial area) and reticular region (deep area). It contains adnexal structures, such as sweat glands, hair follicles, sebaceous glands, and nails. The principal component of the dermis is collagen, particularly type I collagen, which is the major stress-resistant substance of the skin.

The hypodermis, or subcutaneous tissue, consisting of loose connective tissue and adipose tissue, attaches skin to the underlying muscles and bones. Here, the main cell types are fibroblasts, macrophages, and lipocytes. The hypodermis also contains larger blood vessels and nerves which supply the skin.

### 2.2 The Effect of CAP on Skin Cells

There have been several *in vitro* studies in the last few years focusing on the effect of CAP exclusively on non-diseased skin cells (**Table 1**). These projects mostly used HaCaT human immortalized keratinocytes as the cellular model. Although there were some variations in plasma-induced cellular changes, it was found that the respective change at the cellular level was also reflected correspondingly at the protein level and gene level.

**Table 1:** The effect of CAP on skin cells.

Novelty	Cell type	Cellular level	Molecular level	Gene level	Refs
skin renewal activity ***via*** activation of β-catenin	HaCaT keratinocytes	increases cell proliferation *via* cell cycle change	translocation of β-catenin to nucleus	enhances gene expression of c-MYC & cyclin D1	(25)
CAP modulates p53 & activates MAPK signalling	HaCaT keratinocytes	reduction in cell proliferation, transient increase in cell migration	induces accumulation & nuclear translocation of p53	upstream ATM & ATR, downstream MAP, Hsp27, IL-6 & IL-8	(26)
transcriptomic microarray, periodic long-term Tx with PAM	HaCaT keratinocytes	reduces cell motility & induces morphological changes	selective protein validation	260 genes in inflammation & redox homeostasis	(27)
role of ambient gas composition on CAP-elicited cell signalling	HaCaT keratinocytes	O_2_ shielding provides stronger apoptotic effects than N_2_, & induces cell response more efficiently	modulates cell-signalling molecules, alters ROS & RNS compositions in liquids	pathway-specific microarray, IL-6, HMOX1, VEGFA, HBEGF, CSF2, & MAPK	(28)
CAP promotes cell growth ***via*** NF-κB signalling pathway	L929 fibroblasts	increases cell proliferation *via* cell cycle change	secretion of EGF & TGF-β1, upregulates P-p65 & cyclin D1, but downregulates IkB	N/A	(29)
activation of antimicrobial peptides of β-defensin family	primary skin keratinocytes	does not modulate cellular proliferation, migration & apoptosis	induces IL-8, activates TGF-β, promotes β-defensins	induces key regulators	(30)

(ATM, ataxia-telangiectasia mutated; ATR, ataxia telangiectasia and Rad3-related; CAP, cold atmospheric plasma; CSF, colony stimulating factor; EGF, epidermal growth factor; HBEGF, heparin binding EGF-like growth factor; HMOX1, heme oxygenase 1; HSP, heat shock protein; IkB, inhibitor kappa B; IL, interleukin; MAPK, mitogen-activated protein kinase; N_2_, nitrogen; NF-κB, nuclear factor-kappa B; O_2_, oxygen; PAM, plasma-activated medium; RNS, reactive nitrogen species; ROS, reactive oxygen species; TGF, transforming growth factor; Tx, treatment; VEGF, vascular endothelial growth factor).

In a relatively early study, Arndt et al. used the MicroPlaSter β plasma torch system on primary human skin keratinocytes (30). Their main novel finding was that antimicrobial peptides of the β-defensin family were upregulated after CAP treatment. Defensins are small cysteine-rich cationic proteins that are active against bacteria, fungi and viruses. They could be found in inflammatory skin conditions such as atopic dermatitis. CAP also induced gene expression of key regulators for inflammation and wound healing, such as interleukin 8 (IL-8) and transforming growth factor beta (TGF-β). However, the proliferation, migration and apoptosis of keratinocytes were not altered by CAP. Subsequent studies using HaCaT cells found different results, as cell proliferation, motility and apoptosis could all be changed using CAP treatment (25–28).

Choi et al. used a low-frequency argon plasma device based on the dielectric barrier discharge (DBD) technology (25). At the cellular level, keratinocyte proliferation was stimulated by CAP, as the transition from G1 cell cycle phase to S and G2 phases was quickened. At the protein level, CAP not only dispersed E-cadherin-mediated cell-to-cell interactions but also translocated β-catenin from the cytosol to the nucleus. At the gene level, CAP treatment increased the expression and transcription of c-MYC and cyclin D1. Therefore, these results of enhanced epidermal cell growth demonstrated that CAP might be used a novel skin regenerating apparatus.

On the contrary, a series of studies conducted by Schmidt et al. using kINPen 09 atmospheric argon plasma jet showed suppressive results instead. Firstly, reduction in cell proliferation, transient increase in cell migration, and secretion of immunomodulatory signal proteins were found in human keratinocytes after direct CAP exposure (26). The above changes were centred around the p53 regulatory axis, with upstream phosphorylation of ATM and ATR and downstream activation of checkpoint kinases Chk and mitogen-activated protein (MAP) kinases. Thus, plasma-induced proapoptotic, proinflammatory, and pro-survival effects were confirmed. Secondly, periodic, long-term, and indirect treatment using plasma-activated medium (PAM) repressed keratinocyte motility and enlarged cell size (27). The authors discovered differential expression of 260 genes involved in inflammation and redox homeostasis using transcriptomic microarray. The protein products of these deregulated genes include various cytokines, growth factors, antioxidant enzymes, and apoptotic signalling targets. Lastly, the same research group changed reactive species generation of the plasma effluent by modulating the ambient gas composition from pure nitrogen to pure oxygen (28). The oxygen-shielding plasma provided stronger apoptotic effect than the nitrogen counterpart and induced keratinocyte response more efficiently. Gene expression analysis revealed induction of signalling and communication proteins such as immunomodulatory interleukin 6 (IL-6) and several antioxidative molecules.

In summary, the inconsistency in plasma-induced cellular change in healthy skin cells, such as cell proliferation, cell migration, and cell apoptosis are multifactorial. CAP-generating technology (e.g., DBD or others), treatment modality (e.g., direct jet or indirect PAM), plasma parameters (e.g., duration and gas composition), and target skin cell type (e.g., keratinocytes vs. fibroblasts, human vs. murine) all could be contributing factors. More dedicated studies are required to expand our understanding on the effect of CAP on healthy skin cells.

### 2.3 The Effect of CAP on Skin Histology

The above CAP-induced cellular effects have been further examined at the histological level. Additional architectural information was obtained using various histological staining methods including haematoxylin and eosin (H&E) staining, Masson’s trichrome staining, and immunohistochemical (IHC) analysis. The two consecutive studies published by Choi et al. revealed that short period of CAP treatment on HRM2 mice caused significant increases in epidermal thickness and dermal collagen density (25, 31). Repeated CAP treatment not only caused epidermal expansion by activating β-catenin in the epidermal cells, but also accelerated dermal remodelling. On the other hand, plasma increased the skin tissue expression of various growth factors, such as TGF, vascular endothelial growth factor (VEGF), granulocyte macrophage-colony stimulating factor (GM-CSF), and epidermal growth factor (EGF). Interestingly, Hasse et al. brought more clinical value as they used human skin biopsies from nine patients for plasma treatment (32). Unlike what was shown previously where plasma-generated species only penetrated the first few layers of the epidermis (33), the pro-proliferative effect of CAP in the current work occurred as deep as the stratum basale, while the epidermal integrity and keratin expression remained unchanged. Collectively, these *in vivo* studies support CAP as an innovative tool with anti-aging potential for the skin.

### 2.4 The Effect of CAP on Cutaneous Blood Vessels

In general, CAP can improve cutaneous microcirculation of intact skin. In 2016, Kisch et al. organized a controlled, prospective cohort study including 20 healthy patients using skin at the radial forearm (34). Their DBD CAP device increased cutaneous tissue oxygen saturation by 24% and maintained effective for at least 8 min. In addition, the cutaneous capillary blood flow increased by 73% and remained fast for 11 min. Furthermore, even more prominent elevation of these parameters was found in patients with lower body mass index (BMI).

In comparison, Borchardt et al. conducted a similar study with a smaller number of patients, but assessing lot more circulation parameters for a longer monitoring period (35). Firstly, tissue oxygen saturation and blood flow both improved in a plasma duration-dependent manner. Secondly, skin pH decreased by 0.3 after CAP treatment, whereas skin temperature and moisture were not altered. Most importantly, CAP-related enhancement of skin microcirculation was found to be specific to the plasma treatment and not a result of the applied mechanical pressure.

In order to study the influence of CAP on vascularization-involved molecules in skin-related cells, Arndt et al. performed *in vitro* and *in vivo* experiments on a microscopic scale using the MicroPlaSter β plasma torch system (36). CAP significantly activated the expression of artemin, EGF, endocrine-derived VEGF (EG-VEGF), endothelin-1, basic fibroblast growth factor (bFGF), IL-8 in human epidermal keratinocytes, angiogenin, endostatin, monocyte chemoattractant protein (MCP)-1, matrix metalloproteinase (MMP)-9, tissue inhibitor matrix metalloproteinase (TIMP)-1, and VEGF in human dermal fibroblasts, as well as angiopoietin (Ang)-2, angiostatin, amphiregulin, endostatin, bFGF and angiogenic receptors in human umbilical vein endothelial cells (HUVECs). It was concluded that CAP modulates angiogenesis-related factors in an autocrine and paracrine mode.

### 2.5 The Effect of CAP on Skin Chemical Components

The lipids of the stratum corneum is one of the most important components of the skin barrier. Marschewski’s group was one of the first to use X-ray photoelectron spectroscopy (XPS) to analyse CAP-induced changes in the physiological skin lipid composition (37). Using stripped off skin lipids from human forearms, the authors revealed that the total carbon amount reduced from 84.4% to 76.7, oxygen increased from 10.8% to 16.5%, and nitrogen marginally increased from 4.8% to 6.8%. These chemical changes were due to reduced C-C bonds and increased C-O, C=O, C-N, and N-C-O bonds. This proof-of-principle investigation was consolidated by Striesow’s *in vitro* experiment in which collected human forehead lipids were treated using an argon plasma jet (38). The direct-infusion high-resolution tandem mass spectroscopy (DI-MS^2^) and liquid chromatography-tandem mass spectroscopy (RP-LC/MS^2^) detected minimal CAP-driven oxidation of triacyclglycerols, ceramides, and cholesteryl esters. In functional term, epidermal lipid overlay could be well protected, and moderate CAP treatment would result in limited negative consequence in the dermal tissue. In addition, Schmidt et al. conducted an *in vivo* study on murine skin and discovered oxidative modification in the relative abundance of lipid classes using reversed-phase liquid chromatography/mass spectroscopy (39). Finally, Kartaschew et al. used infrared and Raman vibrational microspectroscopy to chemically analyse the plasma-induced change in skin components, such as keratin and lanolin which resembles human sebum (40). The authors suggested that the resultant acidic and NO-containing functional groups could be the source of an antibacterial and regenerative environment in the stratum corneum where plasma interacted with.

Although the aforementioned investigational hardware, such as various types of mass spectroscopy, have greatly facilitated the study of the effect of CAP on skin chemistry, limitations of the analytical software’s ability to identify unexpected oxidized lipids could lead to an underestimation of CAP’s impact on skin lipids (38), suggesting a need for software advancement.

## 3 Treatment of Skin Disease Using CAP

The dermatological applications of CAP can be categorized according to the types of target skin disease, including wound healing, inflammatory skin disorder, infectious skin disease, parasite infestation, skin malignancy, genetic skin disorder, and alopecia (**Table 2**). The studies summarized here are comprised of human clinical trials, preclinical animal work, and laboratory cellular experiments.

**Table 2:** Skin disease-specific application of CAP.

Category	Subgroup	Disease	Test model	Synopsis	Refs
**Wound healing**	cutaneous wound	acute wound	keratinocytes, fibroblasts, mice	accelerates full-thickness re-epithelialization & gap closure	(41)
			mice	combined Tx of Ar & He plasma needle	(42)
			mice	dual effects, low dose boosts but high dose suppresses	(43)
			mice	differential galectin expression in CAP & electrocoagulation	(44)
			mice	enhances burn wound neovascularisation *via* eNOS signalling	(45)
			mice	Nrf2 signalling & inflammation are key events	(46)
			mice	enhances ECM formation in skin graft recipient site	(47)
			bacteria, mice	PAW inactivates bacteria, promotes healing & suppresses inflammation	(48)
			rats	multimodal Tx with CAP & aFGF for multi-tissue regeneration	(4)
			human	RCT on skin graft donor sites	(49)
		chronic wound	MRSA, P. aeruginosa	multi-device inactivation of multi-microbe	(50)
			fibroblasts, mice	activates genes for key cytokines & growth factors	(51)
			rats	higher mechanical strength of CAP-repaired wound in pressure ulcer	(52)
			human	comparable immediate antimicrobial effect to OCT, but limited coverage	(53)
			human	promotes various types of superficial skin erosion wounds healing	(54)
			human	RCT, 2-min Tx of 2 CAP devices reduce bacterial load	(17)
			human	RCT, higher antibacterial effect, ulcer reduction & applicability, lower pt pain	(55)
		diabetic wound	rats	PAW treats STZ-induced diabetic wound	(56)
			keratinocytes, mice	reduces inflammation & oxidative stress, without systemic toxic effects	(57)
			glycated GPx, mice	increases enzyme & antioxidant activity, and reduces inflammation	(58)
			human, foot ulcer	RCT, accelerates healing by decreasing bacterial load & inflammatory phase	(59)
			human, foot ulcer	RCT, beneficial in wound surface reduction & time to wound closure	(60)
	neural wound		astrocytes	PAM, CNS injury	(61)
**Inflammatory disorder**	dermatitis	allergic contact dermatitis	mice	humidified argon plasma	(62)
		atopic dermatitis	mast cells, mice	PAM inhibits mast cell activation, ameliorates HDM-induced AD	(63)
		diaper dermatitis	human	case report	(64)
	psoriasis		keratinocytes	induces cellular apoptosis & reduces IL-12	(65)
			keratinocytes, mice	inhibits pro-inflammatory molecules & increases PD-L1 expression	(66)
			keratinocytes, mice	exerts antiproliferative & proapoptotic effects	(67)
		palmo-plantar psoriasis	human	PCC instrument, case report	(68)
		inverse psoriasis	human	case reports	(69)
	lichen planus		human, mucosal tissue	decreased T-cell infiltrate & inflammatory markers	(70)
**Infectious disease**	fungal infection	dermatophytosis	fungal isolates	lower sensitivity to bacteria and yeast	(71)
			fungal isolates	He/O_2_ suppresses ergosterol biosynthesis, increases keratinase activity	(72)
			guinea pigs	course of infection was a week shorter and milder	(73)
			fungal isolates, guinea pigs	combined Tx of Ag NPs and CAP, comparable with fluconazole	(74)
		onychomycosis	human, nail plate model	cadaver nails & agarose media	(75)
			human, toenail	good clinical cure rate and high patient satisfaction	(76)
			human	synergistic effect of CAP and NPAR	(77)
		alternariosis	dogs	immunocompromised	(78)
	viral infection	HPV	human	adult case reports	(79)
			human	paediatric case series	(80)
		HSV-1	Vero cells, neuroblastoma cells	low but measurable antiviral effect	(81)
**Infestation**	parasitic bites	pediculosis	human	comb-like plasma device	(82)
		demodicosis	human, mite isolates	potential in papulopustular rosacea	(83)
**Skin malignancy**	melanoma		melanoma cells	SMD plasma induces senescence	(84)
			melanoma cells, mice	flexible capillary CAP slows progression of cancer	(85)
			melanoma cells, mice	comparison of direct & indirect CAP treatment	(86)
			melanoma cells	comparison of target genes by ROS & non-ROS	(87)
			melanoma cells	EM emission from CAP kills *via* transbarrier contactless method	(88)
			melanoma cells	PpIX-loaded polymersome-mediated PDT with CAP posttreatment	(89)
			mice	CAP alone & combined with ECT	(90)
			melanoma cells, mice xenograft	CAP & SN synergistically inhibits *via* HGF/c-MET pathway	(91)
	SCC		HNSCC cells	CAP selectively impairs cancer cells	(92)
			HNSCC cells	reduces cell viability & induces apoptosis	(93)
			oral SCC cells	selective killing effect with NO-induced dysfunction of EGFR	(94)
			oral SCC cells	N_2_ CAP inhibits cell migration & invasion most potently *via* decreased FAK & MMP	(95)
			oral SCC cells	synergistic effect of cisplatin & CAP	(96)
			cutaneous SCC cells	PAM induces apoptosis & decreases proliferation	(97)
			cutaneous SCC cells, mice	CAP limits cancer development	(98)
	BCC		BCC cells	PAM induces apoptosis *via* MAPK pathway	(99)
	premalignant lesions	actinic keratosis	human	case series	(100)
			human	seven patients	(101)
			human	various plasma devices	(102)
			human	clinical & ultrasound evaluation	(103)
**Genetic disorder**	keratinization issue	Hailey Hailey disease	human	case report	(104)
**Alopecia**			human dermal hair follicle papilla cells	PAM regulates cell cycle and proliferation	(105)
			human	long-term, PAM	(106)
			Wistar rats	N_2_ plasma increases hair follicle diameter	(107)
**Symptom of skin disease**	pruritus		human	various aetiologies, VAS similar to Ar	(108)

(AD, atopic dermatitis; aFGF, acidic fibroblast growth factor; Ag, silver; Ar, argon; BCC, basal cell carcinoma; CAP, cold atmospheric plasma; CNS, central nervous system; ECM, extracellular matrix; ECT, electrochemotherapy; EGFR, epidermal growth factor receptor; EM, electromagnetic; eNOS, endothelial nitric oxide synthase; FAK, focal adhesion kinase; GPx, glutathione peroxidase; HDM, house dust mite; He, helium; HNSCC, head and neck squamous cell carcinoma; HPV, human papillomavirus; HSV, herpes simplex virus; IL, interleukin; MMP, matrix metalloproteinase; MRSA, methicillin-resistant staphylococcus aureus; N_2_, nitrogen; NO, nitric oxide; NP, nanoparticle; NPAR, nail plate abrasion & refreshment; O_2_, oxygen; OCT, octenidine; PAM, plasma-activated medium; PAW, plasma-activated water; PDT, photodynamic therapy; PpIX, protoporphyrin IX; pt, patient; PCC, plasma coagulation controller; PD-L1, programmed death-ligand 1; RCT, randomized controlled trial; ROS, reactive oxygen species; SCC, squamous cell carcinoma; SMD, surface micro discharge; SN, silymarin nanoemulsion; STZ, streptozotocin; Tx, treatment; VAS, visual analogue score).

### 3.1 Wound Healing

#### 3.1.1 Cutaneous Wound

CAP can boost skin wound healing by several advantages including its antiseptic effect and pro-angiogenic effect, stimulation of cell proliferation and migration, and modulation of topical inflammation (109). The healing of various types of wounds, such as acute wound, chronic wound, and diabetic wound, can all be promoted using CAP treatment.

##### 3.1.1.1 Acute Wound

Based on the above known phenomena, several studies provided additional mechanistic information regarding CAP treatment on acute skin wound (41, 45, 46). CAP could accelerate wound gap closure through downregulation of connexin 43, E-cadherin, and several integrins, which are involved in adherence junctions and cytoskeletal dynamics (41). CAP could enhance wound neovascularisation through modulating endothelial nitric oxide synthase (eNOS) and VEGF signalling (45). CAP could also balance antioxidant and inflammatory response through regulation of the nuclear E2-related factor (Nrf2) pathway and redox-sensitive p53 signalling (46). On the other hand, few other studies afforded practical cues (42–44). CAP treatment could be customized according to different stages of wound healing, because argon plasma and helium plasma were found to be better suited for coagulation purpose and healing purpose, respectively (42). In comparison to electrocoagulation, CAP’s effect could be monitored using galectins as a marker as they were inhibited in the former treatment modality and increased in the latter (44). Nonetheless, CAP duration needs to be carefully controlled during wound treatment, as overdose of plasma could suppress wound healing due to excessive necrosis or apoptosis (43).

Third-degree burn and full-thickness surgical incision are the commonly used models of acute wound in the above work. Skin grafting is a unique procedure which removes healthy skin from one area of the body and transplants it to a damaged area. In the donor site, Heinlin et al. conducted a randomized placebo-controlled trial and observed improved wound re-epithelialisation and fewer fibrin layers and blood crusts after CAP treatment (49). In the recipient site, Frescaline et al. found that CAP could enhance extracellular matrix (ECM) formation through activating the canonical SMAD-dependent TGF-β1 pathway (47).

Finally, CAP treatment of acute skin wound has proven versatile in terms of its multimodality. In addition to direct plasma jet, CAP could be used indirectly in a form of PAM. Xu et al. discovered that PAM could equally inactivate bacteria, suppress inflammation, and promote wound healing (48). Lately, Tan el al. noticed that CAP could synergistically work with acidic FGF during wound healing and other tissue regenerative processes (4).

##### 3.1.1.2 Chronic Wound

Although a chronic wound starts as an acute wound, it does not progress through the expected stages of wound healing within the predicted timeframe, resulting in a prolonged recovery. Major types of chronic wounds include vascular ulcers, pressure ulcers, and diabetic ulcers which will be discussed separately. They share some common features, such as excessive inflammatory phase, persistent infection, and formation of drug-resistant biofilm (110). CAP’s antimicrobial effect is the most sought after during its chronic wound management.

Firstly, Mohd Nasir et al. found that CAP generated using the parallel plate dielectric barrier discharge (DBD) and the capillary guided corona discharge (CGCD) technology can both inactivate methicillin-resistant *Staphylococcus aureus* (MRSA) and *Pseudomonas aeruginosa*, which are two common bacteria found in clinically recalcitrant chronic wounds (50). Although the antibacterial efficacy of these two plasma designs differed for various strains of the above bacteria. Secondly, Ulrich et al. discovered that the immediate antimicrobial effect of CAP was comparable to octenidine (53), a modern antiseptic for cutaneous and mucous wounds (111). Although both treatments were well tolerated by patients with chronic leg ulcers, octenidine outperformed CAP in the long term. Lastly, two randomized controlled trials (RCTs) established solid antimicrobial effect during CAP treatment for chronic wound. Isbary et al. showed that two-minute argon plasma treatment was sufficient to significantly reduce bacterial load regardless of the species of bacteria (17). Whereas Brehmer et al. demonstrated that plasma treatment not only led to objective reduction in bacterial load and ulcer size in chronic venous leg ulcers, but also subjective elevation in patient and physician satisfaction (55).

In addition to the vascular ulcer model applied in the above experiments, pressure ulcer model was used in Chatraie’s work recently (52). This interesting study focused on the closure effect of CAP for chronic wound. The results showed that the mechanical strength of repaired wound in the plasma-treated group was significantly higher than that in the control. This implied that CAP could offer improved tissue strength and maturity during chronic wound healing.

##### 3.1.1.3 Diabetic Wound

Unlike vascular and pressure chronic ulcers, diabetic wound has higher disease prevalence and distinct pathology, such as neuropathy, microangiopathy, and hyperglycaemia (110). Few animal studies showed consistent findings in diabetic wound healing by CAP, such as improved wound closure and angiogenesis as well as reduced inflammation and oxidative stress (56–58). Moreover, each of these studies provided additional information. Indirect plasma treatment, e.g., PAM, increased the level of antioxidants in wound tissue, such as glutathione peroxidase (GPx) (56), whereas direct plasma treatment of diabetes-induced glycated GPx considerably enhanced its enzyme antioxidant activity (58). Most importantly, CAP treatment did not cause systemic toxic effects based on biochemical results from routine blood tests (57).

#### 3.1.2 Neural Wound

Recent research on CAP-facilitated wound healing has moved from cutaneous tissue to others, such as neural tissue. In a pilot study by Sardella et al., PAM was found to be able to modulate both astrocyte growth and migration as a function of RONS (61). Specifically, H_2_O_2_- and nitrite-enriched PAM elicited negative and neutral effect on cell growth, respectively, while balanced PAM could boost astrocyte growth and ameliorate neural wound healing. This could potentially extend CAP-aided wound healing to central nervous system (CNS) injuries, such as Alzheimer’s disease (AD) and Parkinson’s disease (PD).

### 3.2 Inflammatory Skin Disorder

#### 3.2.1 Dermatitis

Dermatitis is simply inflammation of the skin. Itchiness, redness, and rash could be the symptoms in the early stage of dermatitis, and oedema and blister in the more serious. From a pathophysiological viewpoint, dermatitis is characterized by spongiosis which allows inflammatory cells and mediators to accumulate. Common types of dermatitis include allergic, atopic, and seborrheic dermatitis.

Firstly, using a mouse model of allergic contact dermatitis (ACD), Xiong et al. found that humidified CAP is better than oxygen or nitrogen plasma when treating mild and severe ACD (62). This was mainly because active species delivered by water-containing argon plasma became adhesive to the dry skin of ACD leading to good penetration of ROS. Secondly, Lee et al. discovered that PAM could inhibit mast cell activation by inhibiting the expression of IL-6, tumor necrosis factor (TNF)-α, and nuclear factor kappa B (NF-κB) (63). In addition, direct CAP treatment could ameliorate house dust mite (HDM)-induced atopic dermatitis in mice by suppressing the recruitment of mast cells and eosinophils as well as differentiation of Th2 cells. Finally, in a case report on a 14-month-old girl with diaper dermatitis with urinary and faecal bacterial infection, who failed topical and oral medical treatment, Zhang et al. presented satisfactory therapeutic effect of CAP (64). After six sessions of treatment, patient’s pain and genital ulcer completely resolved without any adverse reactions or disease recurrence.

#### 3.2.2 Psoriasis

Psoriasis is a multisystem autoimmune syndrome that presents with well-defined, pink, itchy scaly patches predominantly on the extensor surface of the body, such as elbows and knees, back, and scalp. The two main pathogenic processes of psoriasis are dermal inflammation and immune suppression, and secondary epidermal proliferation.

In a cellular model of psoriasis, Zhong et al. found that CAP could induce apoptosis, mitochondrial dysfunction, and lysosomal leakage in human keratinocytes, as well as reduce the expression of IL-12 (65). Overall, these effects have implication to control amplification of skin inflammation by suppressing keratinocyte hyperproliferation and targeting T-cell activation.

Using mouse model of imiquimod-induced psoriasis, Gan et al. proved that repeated administration of CAP could ameliorate psoriatic appearance and reduce epidermal proliferation (67). On the other hand, Lee et al. noticed that plasma treatment could inhibit expression of pro-inflammatory molecules and upregulate that of programmed cell death ligand 1 (PD-L1).

In case reports of patients with psoriasis, Gareri et al. and Zheng et al. established satisfactory inhibitory or even curative effect of CAP on palmo-plantar psoriasis and inverse psoriasis, respectively, which are subtypes usually resistant to traditional treatments (68, 69).

#### 3.2.3 Lichen Planus

Lichen planus (LP) is a chronic inflammatory and immune-mediated disorder mainly affecting skin and oral mucosa. Cutaneous LP is characterized by a pruritic, polygonal, purple papule or plaque with overlying whitish striae. Basal cell damage and liquefaction led by T-cell mediated inflammatory infiltrate potentially contributes to the pathogenesis of LP. Seebauer et al. performed an ex vivo experiment using mucosal tissue samples from patients with oral LP, and treated them using CAP (70). The oral LP tissue showed increased infiltrate of CD8^+^ and CD45-R0^+^ T cells, and higher level of IL-1β, IL-6, IL-8, and GM-CSF. CAP treatment of these diseased mucosal tissue significantly reduced the production of inflammatory cytokines and chemokines, and decreased T-cell infiltrate.

### 3.3 Infectious Skin Disease

Most bacterial skin infections are caused by gram-positive organisms, such as *Staphylococci* and *Streptococci*. Since the antibacterial effect of CAP has already been discussed in ‘Cutaneous wound healing’ (section 3.1.1.) and other reviews, treatment of bacterial skin disease will not be elaborated here. More focus is placed on CAP therapy for fungal and viral skin diseases.

#### 3.3.1 Fungal Infection

Dermatophytosis is a fungal infection of the skin caused by dermatophytes which absorb nutrients from keratin in the stratum corneum. It typically manifests as a red, itchy, scaly, and circular rash. Recently, there have been several *in vitro* (71, 72) and *in vivo* (73, 74) studies exploring CAP’s antifungal potential. Scholtz et al. tested CAP on four human pathogenic dermatophytes including anthropophilic *Trichophyton rubrum* and *Trichophyton interdigitale*, zoophilic *Arthroderma benhamiae*, and geophilic *Microsporum gypseum* (71). Although the sensitivity of dermatophytes to CAP treatment seemed to be genus-specific and lower than that of bacteria and yeast, 25-min of CAP exposure could cause complete fungal spore inactivation and thus decontamination. In addition, Shapourzadeh et al. used helium/oxygen (98%/2%) CAP on *Trichophyton rubrum* (72). CAP treatment significantly inhibited fungal growth by up to 91%, reduced ergosterol biosynthesis by up to 54%, and increased keratinase activity by up to 21% in a plasma dose dependent manner. Again, Scholtz et al. tested CAP on guinea pigs that were infected with *Trichophyton mentagrophytes* SK 3286 dermatophyte. Their customized plasma device not only lowered the colony-forming unit (CFU) of fungal counts, but also made the course of infection a week shorter and milder. Finally, Ouf et al. combined CAP treatment with silver nanoparticles (AgNPs) and applied it on an animal model of dermatophytosis (74). Combinatorial therapy was found to be better than standalone treatment, and comparable to fluconazole in terms of antifungal efficacy.

Onychomycosis is a fungal infection of the nail plate and the nail bed, and more commonly found in toenails than in fingernails. Its symptoms include discoloured and thickened nail plate, subungual debris, and onycholysis. In an *in vitro* study using a novel toenail-plate model using cadaver nails and fungi-inoculated agarose mould, Bulson et al. found that direct CAP exposure resulted in complete killing of *Candida albicans* and *Trichophyton mentagrophytes* at a low dose, and CAP treatment through the nail plate reduced viability of *C albicans* at a higher dose (75). In 2017, Lipner et al. recruited 19 patients with toenail onychomycosis. The overall clinical cure and mycological cure were 53.8% and 15.4%, respectively, after CAP treatment (76). In 2020, Lux et al. significantly improved the above indices to 70% and 85.7% using the synergistic combination of CAP and nail plate abrasion and refreshment (NPAR) (77).

#### 3.3.2 Viral Infection

Studies regarding CAP’s antiviral potential only started two years ago, and the number of relevant works is very limited. Herpes simplex virus (HSV) is common and contagious, and its mode of transmission include direct contact of skin or mucosa, causing watery blisters in these locations. HSV-1 produces most herpes labialis but can also cause conjunctivitis and encephalitis. Bunz et al. applied HSV-1 infection to a standard HSV research cell line, Vero cells, and the neuroblastoma cell line SH-SY5Y as a model for neuronal infection (81). They revealed a low but measurable antiviral effect of CAP on HSV-1.

Human papilloma virus (HPV) causes warts on the skin or mucous membrane, which are thought to arise from the proliferation of infected basal keratinocytes. Friedman’s group recently published two small case series including two adult patients (79) and five paediatric patients (80), both of which reported remarkable clearance of verruca vulgaris, periungual warts and palmoplantar warts.

### 3.4 Parasitic Infestation

Demodicosis in humans is usually caused by *Demodex folliculorum* mites, which may be a cause or exacerbating factor in papulopustular rosacea. Rosacea is a chronic and disfiguring condition that typically affects the midfacial skin. Daeschlein et al. successfully used CAP *in vitro* as a non-antimicrobial treatment to inactivate *Demodex folliculorum* isolated from a patient (83). CAP’s antiparasitic efficacy could suggest an alternative to Rosacea treatment.

Pediculosis is an infestation of lice including three types, namely pediculosis capitis (head louse infestation), pediculosis corporis (body louse infestation), and pediculosis pubis (pubic louse infestation). Ten Bosch et al. developed a comb-like CAP device designed as a physical remedy for pediculosis capitis (82). Their results showed that a single stroke of plasma over a hair strand led to high mortality rates of *P. humanus humanus* and parasitic eggs. The safety of this novel insecticide-free option was also verified in three aspects with satisfaction, e.g., ozone concentration, UV emission, and patient leakage current.

### 3.5 Skin Malignancy

Skin cancer include three main types, i.e., basal cell carcinoma (BCC), squamous cell carcinoma (SCC), and melanoma, which is in decreasing order of prevalence and increasing order of aggressiveness. One of the foundations for plasma oncology is CAP’s ability to selectively kill cancer cells over surrounding healthy cells (112). Most of recent relevant work are cellular and animal studies.

#### 3.5.1 Melanoma

Melanoma develops from melanocytes and is the most dangerous type of skin cancer. Its local invasiveness is contributed by both horizontal and vertical spread, e.g., into the epidermis or hypodermis. Warning signs of malignant melanoma include asymmetry, borders (irregular), colour (variegated), diameter (> 6 mm), and evolving.

Standalone CAP treatment has been proven effective in inhibiting melanoma cells. Arndt et al. discovered differential induction of apoptosis or senescence of melanoma cells in respond to different CAP does using a novel CAP device based on the surface micro discharge (SMD) technology (84). Binenbaum et al. adjusted the conventional plasma jet system and developed a hand-held elongated flexible capillary to deliver CAP to the target tumor (85). Tumor volume of cutaneous melanoma was significantly reduced by the above treatment, in a dose dependent fashion. In Saadati’s interesting work, the antineoplastic effect of direct and indirect CAP treatment for melanoma was compared *in vitro* and *in vivo* (86). Although the direct plasma treatment was more effective than the indirect one, PAM when combined with chemotherapeutic drug was more potent than the direct mode.

Therefore, increasing number of studies dedicated to combining CAP with other treatment modalities, such as electrochemotherapy (ECT) (90), photodynamic therapy (PDT) (89), and nanomedicine (91). Using a melanoma mouse model, Daeschlein et al. proved that CAP combined with ECT significantly suppressed tumor growth acceleration and daily volume progression, and improved mouse survival. Wang et al. loaded protoporphyrin IX into polymersomes and achieved 50% killing of melanoma cells *via* PDT. With the application of CAP posttreatment as a novel light source, melanoma cell viability decreased even more. In addition, Adhikari et al. realized that co-treatment of CAP and silymarin nanoemulsion could synergistically inhibit melanoma tumorigenesis *via* targeting HGF/c-Met pathway in human melanoma cells and mice xenografts.

Recently, some researchers revisited CAP’s antitumor potential from a different perspective, i.e., non-RONS component of CAP. Yan et al. demonstrated that the electromagnetic (EM) emission from CAP could lead to the death of melanoma cells *via* a transbarrier contactless method (88). Compared to RONS, EM elicited stronger inhibitory effect on a reactive species-resistant melanoma cell line B16F10, owing to a new course of cell death. On the other hand, Ji et al. performed a genome-wide comparison of the target genes of the ROS and non-ROS constituents of CAP in cancer cells including melanoma cells (87). The authors identified CAP-specific genes governed by constituents other than RONS.

#### 3.5.2 Squamous Cell Carcinoma

Cutaneous SCC is a malignant tumor of keratinocytes that infiltrates the dermis, and commonly presents as a red, crusted and scaling patch, or firm and hard nodule. Microscopically, there is cellular and nuclear heterogeneity, focal and abnormal keratinisation, and often invasion of surrounding tissue.

Several studies have established CAP’s selectivity for SCC cells over healthy cells, each with distinct extra findings (92–94, 98). Guerrero-Preston et al. suggested that helium CAP selectively impaired head and neck SCC cells through non-apoptotic mechanism (92). Welz et al. demonstrated that SMD CAP could generate anticancer effects that have been previously shown in other studies using DBD-based plasma (93). Lee et al. took a step further by conducting mechanistic study for CAP’s selectivity (94). They found that degradation and dysfunction of epidermal growth factor receptors (EGFRs) were observed only in EGFR-overexpressing SCC and not in the normal human fibroblasts after CAP treatment. In addition, nitric oxide (NO) scavenger pre-treatment rescued above EGFR abnormality. Thus, CAP might be a promising alternative remedy for SCC by inducing EGFR dysfunction *via* NO radicals. Meanwhile, Pasqual-Melo et al. verified CAP’s selectivity *in vivo*, and found that CAP limited the progression of SCC without compromising non-SCC skin, with the expression of antioxidant transcription factor Nrf2 reduced in the lesion and increased in the non-lesion tissue (98).

Apart from being selective for SCC, CAP has recently demonstrated its technical versatility during SCC treatment (95–97). Firstly, indirect CAP management, such as PAM, had a prominent anticancer effect on cutaneous SCC cells (97). Secondly, Lee et al. showed synergistic effect of CAP and cisplatin chemotherapy against SCC cells (96). Finally, by optimising the plasma gas type, Kang et al. revealed that nitrogen CAP inhibited SCC cell migration and invasion most potently through reduced expression of focal adhesion kinase (FAK) and MMP (95).

#### 3.5.3 Basal Cell Carcinoma

BCC is a locally invasive but rarely metastasizing skin cancer of basaloid cells. A typical nodular BCC is characterised by pearly and translucent appearance with telangiectasia, and occasionally central ulceration. Yang et al. demonstrated that PAM, e.g., CAP-activated phosphate-buffered saline (PBS) and Dulbecco’s modified Eagle’s Medium (DMEM), could inhibit cell viability and promote cell apoptosis of BCC cells *in vitro* (99). Using next-generation RNA sequencing, the authors manifested upregulated MAPK, TNF and IL-17 signalling pathways, which might be strongly associated with PAM-induced apoptosis.

#### 3.5.4 Premalignant Lesions

Actinic keratosis (AK) is the most common precancerous condition found in skin that’s damaged by chronic solar or ultraviolet exposure. It tends to appear as thick, scaly, or crusty plaques that are better felt than seen. On histopathological examination, AK usually shows atypical keratinocytic proliferation begging in the basal layer and confined to the epidermis.

Recent case series published by two independent research groups both presented successful treatment of AK using CAP (100, 101). Subsequently, Friedman et al. summarized the main differences between these two studies, including number of lesions treated, regime of plasma treatment, and grading system used for treatment measurement (102). The authors proposed that treatment efficacy should be evaluated by their success of full AK elimination rather than just reduction in their clinical thickness. Last year, Arisi and co-workers conducted a larger study using CAP for the treatment of AK and skin field cancerization (103). It was found that plasma irradiation was effective for reducing the cumulative AK area, the actinic keratosis area and severity index (AKASI) score, and the number of AKs. In addition, high-frequency ultrasound showed that CAP treatment improved features of chronic photodamage of the skin underlying and surrounding the AK spots.

### 3.6 Hair Loss

In an *in vitro* cellular study, Lee et al. manipulated cell cycle progression of human hair follicle dermal papilla cells (DPCs) using CAP-activated medium (105). Human DPCs are mesenchymal cells isolated from the hair papilla of healthy scalp hair follicles. In the adult hair follicle, hair papilla plays a key role in controlling hair production and hair growth (113). PAM treatment at an appropriate concentration and duration was found to arrest cell proliferation and cell cycle of DPCs, which could gradually recover.

In an *in vivo* animal study using Wistar rats, Babossalam et al. achieved skin rejuvenation using a novel pulsed nitrogen plasma touch (107). A significant increase in epidermal thickness, fibroblast proliferation, and skin collagenesis were discovered. Most importantly, CAP also increased the diameter of primary and secondary hair follicles in the treated skin.

In a human trial including 14 patients with androgenic alopecia, Khan et al. tested long-term CAP treatment in the form of PAM (106). The theoretical bases for this study were CAP’s ability to induce stem cell differentiation in various cell types (3), and its inconsistency in terms of depth of penetration, especially on thick human scalp skin. The six-month periodic PAM washing was well tolerated, and most patients and clinicians reported subjective and qualitative improvement.

## 4 Improving Drug Absorbance Into Skin Using CAP

Transdermal drug delivery (TDD) is a method to deliver topical medications through the stratum corneum into the deeper skin and blood circulation for local and systemic therapeutic purpose. Several strategies have been developed, such as iontophoresis, electroporation, and microneedles, to surmount the barrier for drug penetration. CAP could overcome the limitations of the above strategies, and serve as an non-invasive and efficient pre-treatment to improve TDD (114). The exact mechanism is multifactorial. Firstly, CAP could enhance the transdermal permeation of drugs, such as galantamine hydrochloride (115) and cyclosporine (116). Secondly, CAP could modify the composition of skin lipids, which has been discussed in section 2.5 and elsewhere (117, 118). Since the keratinocytes and intercellular lipid matrix compose the stratum corneum, CAP therefore could change the function of skin barrier (section 2.1). Thirdly, CAP could downregulate the expression of E-cadherin, which temporarily impairs the tight junction opening up intercellular pores (119). Recent advances on CAP-facilitated TDD focused on two aspects: manipulating plasma parameters as an upstream approach and testing new drugs as a downstream approach.

In two consecutive studies by Gelker et al., the electrical characteristics of plasma could be tailored to cause differential permeabilization of excised human full-thickness skin (120, 121). Both μs-pulsed and ns-pulsed DBD plasma could significantly reduce the transepithelial electrical resistance (TEER). In addition, the μs-pulsed CAP induced more prominent pore formation in stratum corneum and skin permeabilization of a test drug, comparing to the ns-pulsed DBD. Increasing the power of plasma led to more pronounced results but might increase the risk of skin side effects, highlighting the importance of safety and risk assessment before clinical application of CAP-aided TDD.

In another consecutive studies by Xin et al., transdermal delivery of a local anaesthetic drug, lidocaine, was enhanced by CAP pre-treatment (122, 123). Using a mouse model, the authors found that the transdermal flux of lidocaine was nearly doubled, and the drug penetration reached deeper parts of skin. The transepidermal water loss (TEWL) value increased dramatically right after CAP treatment and recovered in the next 3 hours, indicating a transient and reversible change in the skin barrier function. Using a randomized split-face study including 20 patients, the authors further proved a satisfactory and enhanced analgesic effect of topical lidocaine after CAP pre-treatment. The visual analogue scale (VAS) for pain was statistically lower in the CAP group than in the control. Most importantly, apart from some minor disturbance in skin sensation, no severe adverse events were noted.

## 5 Safety and Risk Assessment in Plasma Dermatology

Although CAP has been proven as a promising treatment modality for clinical dermatology as explained in the above sections, the safety aspects must be carefully examined prior to its implementation. Due to the complexity of CAP constituents, such as electrons, ions, RONS, electromagnetic emission, and UV radiation, plasma overtreatment is very likely to cause undesired side effects, such as sensory disturbance, physical damage and thermal injury (33, 123, 124).

Short-term safety analysis focused on the effect of plasma factors. Firstly, Daeschlein et al. investigated the risks of three different plasma sources, i.e., pulsed plasma jet, non-pulsed plasma jet, and DBD plasma (125). All plasma treatments were well tolerated and did not damage the skin barrier nor cause skin dryness. Secondly, Wiegand et al. discovered dose- and time-dependent cellular effects of CAP using a 3D skin model (126). Increasing plasma input power or treatment intervals led to detrimental effects, and air as working gas was more damaging than nitrogen. Finally, Kos and co-workers did further dissections of plasma overtreatment-related skin damage (127). The direct skin damage, as a result of released heat and RONS, deteriorated as treatment time increased and gas flow rate rose, and mainly manifested as skin burn. On the contrary, the indirect skin damage, presented as local oedema, was only noticed after two days of plasma overtreatment, and plasma parameters independent.

The results of long-term safety analysis of CAP treatment were encouraging. Schmidt et al. investigated the long-term side effects of repetitive argon plasma treatment over 14 consecutive days in a rodent model with full-thickness skin wound (128). After one year, blood tests, quantitative PCR, and IHC analysis all failed to reveal any systemic or local side effects, such as chronic inflammation or tumor formation. Furthermore, Rutkowski et al. completed a 5-year clinical follow-up of five patients who originally participated in a study of wound healing using CAP treatment (129). A complex imaging diagnostic assessment did not show any signs of malignant tumor, inflammatory reaction, or pathological change in the plasma-treated areas.

In summary, CAP treatment for normal and diseased skin is generally safe, provided excessive plasma exposure is avoided. Due to the significant variation in plasma devices and their operational parameters, the exact cut-off in plasma dose between safety and risks cannot be determined just yet. Further clinical studies are required to clarify indication-specific dose recommendation.

## 6 Conclusion and Future Opportunities

Conventional treatment modalities in clinical dermatology all have limitations and drawbacks. Cold atmospheric plasma has demonstrated excellent potential partially owing to its technical versality, such as direct irradiation of superficial lesions, indirect treatment of deeper and larger disorders using plasma-activated medium, and co-treatment with CAP and other therapeutic methods. Apart from treatment of skin diseases, CAP can also optimise intact skin and facilitate transdermal drug delivery with controllable safety and minimal side effects. Nonetheless, several challenges remain to be fully addressed before we can apply this unique therapy in daily clinical practice.

Firstly, the number of CAP-treatable skin diseases covered here are still quite limited. To our knowledge, there has been no substantial research on the treatment of acne, urticaria, blistering disorders, and benign skin tumours using CAP so far. Secondly, treatment of full-body skin conditions, such as autoimmune disorder, systemic vasculitis, acquired immune deficiency syndrome (AIDS), and metabolic disease, might be technically problematic and time-consuming for CAP. Thirdly, the quality of relevant studies remains suboptimal. There is a rapidly growing demand for randomized double blind placebo control studies on CAP-aided skin treatment. In order to encourage CAP to realize its full potential in dermatology, an efficient collaboration and proactive communication between clinical dermatologists and plasma biotechnologists need to be secured.

## Author Contributions

FT: conceptualization, data curation, formal analysis, funding acquisition, investigation, methodology, project administration, resources, software, supervision, validation, visualization, writing. YW: resources, visualization. SQZ: investigation, methodology. RYS: resources, visualization. JHC: data curation, methodology, resources, visualization. All authors contributed to the article and approved the submitted version.

## Funding

This work is sponsored by the Fundamental Research Funds for the Central Universities.

## Conflict of Interest

The authors declare that the research was conducted in the absence of any commercial or financial relationships that could be construed as a potential conflict of interest.

## Publisher’s Note

All claims expressed in this article are solely those of the authors and do not necessarily represent those of their affiliated organizations, or those of the publisher, the editors and the reviewers. Any product that may be evaluated in this article, or claim that may be made by its manufacturer, is not guaranteed or endorsed by the publisher.
